# A Miniaturized Ultrawideband V-Shaped Tip E-Probe for Near-Field Measurements

**DOI:** 10.3390/s24134295

**Published:** 2024-07-02

**Authors:** Mahmoud Mohammed Khodeir, Zhaowen Yan, Fuyu Zhao

**Affiliations:** School of Electronic and Information Engineering, Beihang University, Beiijng 100191, China; mahmoud1985@buaa.edu.cn (M.M.K.); by2302128@buaa.edu.cn (F.Z.)

**Keywords:** miniaturized, ultrawideband, electric field probe, high sensitivity, high spatial resolution, near-field measurement

## Abstract

A sensitive, miniaturized, ultrawideband probe is proposed for near-field measurements. The proposed probe is based on a new V-shaped tip design and a slope structure resulting in better field distribution and impedance matching with a span bandwidth from 10 kHz up to 52 GHz, which is compatible with ultrawideband applications. The proposed E-probe fabrication process utilizes a four-layer printed circuit board (PCB) using Rogers RO4003 (tm) and RO4450 high-performance dielectrics, with εr = 3.55 and 3.3, respectively. The probe length is 40 mm with a minimum width of 4 mm, which is suitable for narrow, complex, and integrated PCBs. The passive E-probe sensitivity is −106.29 dBm and −87.48 dBm at 2 GHz and 40 GHz, respectively. It has a very small spatial resolution of 0.5 mm at 20, 25, 30, and 35 GHz. The probe is small and cheap and can diagnose electromagnetic interference (EMI) in electronic systems such as telemetry, UAVs, and avionics.

## 1. Introduction

Currently, electromagnetic compatibility (EMC) is one of the main methods for measuring the ability of electric and electronic devices to work properly in the surrounding environment. Currently, electromagnetic compatibility is indispensable in the electrical and electronic industries, and electronic circuit development is growing toward high-speed and high-frequency circuit technologies, resulting in very small and complex integrated circuits. Due to this rapid growth, the problem of electromagnetic interference (EMI) has become obvious and serious, and the electronics industry has paid attention to overcoming this problem. The problem of the electromagnetic interference (EMI) has a serious effect on the performance of high-speed circuits and, therefore, leads to the abnormal function of the high-speed circuits. To protect our electronic systems environment, the electromagnetic interference (EMI) source must be determined or detected. In addition, all electronic devices must be exposed to the electromagnetic compatibility (EMC) test before being released to the marketplace. Various EMC standards have been established globally for product performance testing. To meet the stringent EMC standards, more attention has been given to mitigating EMI problems, which may require additional design considerations and filtering techniques [1,2,3,4,5].

Electromagnetic compatibility (EMC) and electromagnetic interference (EMI) mainly depend on near-field (NF) measurement scanning, so there are many different techniques used for near-field scanning, such as fiber-based electro-optic NF techniques [6,7,8], solid-state quantum NF techniques [9,10], and electromagnetic probe techniques.

This paper is interested in the electromagnetic probe techniques; so, based on Maxwell’s equations, electromagnetic probes are the main tool for pointing and locating the source of electromagnetic interference (EMI). The probes rapidly locate the EMI source by sensing the near-filed emissions from the device under test (DUT); in addition, it is a cost-effective tool that can be widely used in near-field measurements. Moreover, the International Electrotechnical Commission IEC has published the IEC TS 61967-3:2014 standard [11,12], which mentioned the use of electromagnetic probes as a standard tool for the near-field measurement of the electric and magnetic field components emitted from or near any printed circuit board (PCB) and stipulated the test procedures and technical specification for detecting and measuring the near-electric, magnetic, or the electromagnetic field at or near any printed circuit board (PCB) or integrated circuit (IC), which can be divided into two main types—electric near-field probes (E-probes) [13,14,15,16] and magnetic near-field probes (H-probes) [17,18,19,20]. Magnetic probes depend on the current induced in the loop tip of the probe due to the magnetic field generated around the tested device (device under test—DUT) according to Faraday’s law. Electric probes usually depend on the induced current in the probe tip due to the electric field radiated from the device under test (DUT) in the z-direction, perpendicular to the substrate; E-probes are monopoles and the induced current is measured according to Maxwell’s displacement current theory.

Earlier electric field probes were designed using coaxial methods, which were optimized for operating below the 20 GHz frequency range. For enhancing the working bandwidth, controlling the impedance, and accuracy, the current academic research is interested in the stripline (SL) electric probe structure, which consists of three main parts—the induction part, the transmission part, and the output part—which can work at up to 60 GHz.

In this paper, an ultrawideband electromagnetic probe is first introduced, with a working frequency from 10 kHz to 50 GHz, with a stripline (SL) structure working for a real-time scanning and monitoring system. The paper’s methodology for the novel V-shaped tip probe structure can be introduced starting from the basic idea of the probe’s working principal and the basic idea for the enhancement of the induced capacitance between the DUT and the probe tip; after that, the simulation, fabrication, measurement, and probe application are described.

## 2. Proposed E-Probe Design

### 2.1. Probe Design Overview

**Figure 1:**
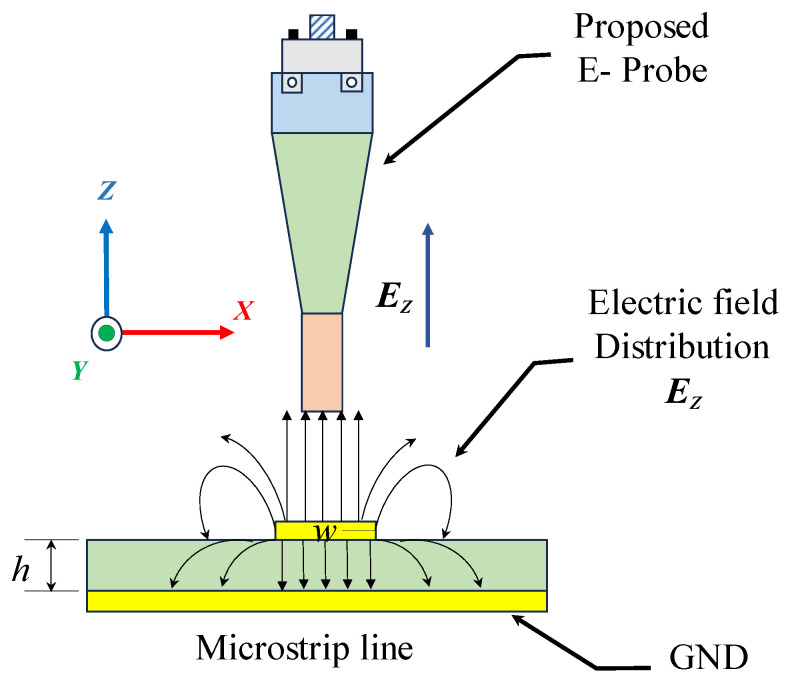
Simplified diagram for the electric field generated from the microstrip line.

The proposed electric field probe is designed to detect the z-direction component of the electric field generated from the device under test (DUT). As shown in Figure 1, the distribution of the electric field in the microstrip line is perpendicular to the substrate surface and incident to the probe tip.

**Figure 2:**
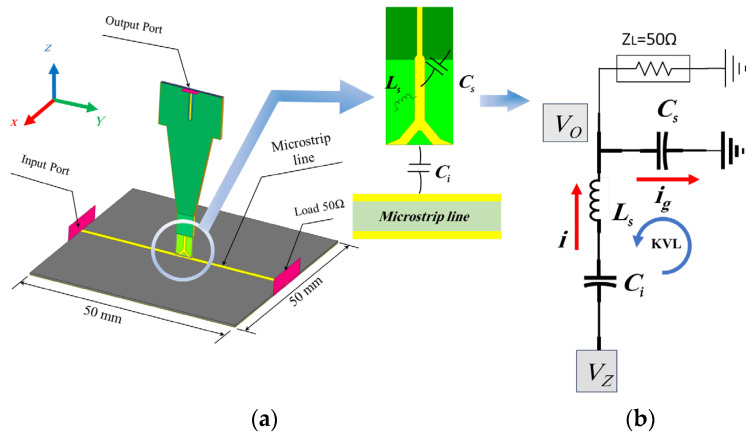
(**a**) Model of the proposed E-probe. (**b**) Equivalent circuit for the electric field above the DUT.

Figure 2 shows that the device under test (DUT) is a two-layer printed circuit board (PCB) with a dimension of 50 mm × 50 mm. A substrate of Rogers RO4003 (tm) with εr = 3.55, height (h) = 0.508 mm, a dielectric loss tangent of 0.0027, and a microstrip line width (w) of 1.16 mm is excited using a signal generator with a frequency of 10 GHz, and is matched using a 50 Ω load. The dominant electric field is E_Z_ component, which is distributed perpendicular to the substrate and incident to the probe tip.

### 2.2. Probe Analysis and Equivalent Circuit

The E-field probe operates on the principle of a capacitive probe when exposed to an electromagnetic field, whereby the electric current is induced within the capacitance *Ci*. This capacitance is the key point of the induction part and the coupling process of the working theory of the E-field probe, and the probe will be placed perpendicular to the microstrip, as shown in Figure 2a, and will detect the E-field. According to [21], the induced current *i* in the probe tip can be calculated as follows:

(1) $$i=C\;\frac{\mathrm{du}\left(t\right)}{\mathrm{dt}}$$

so,

(2) $$u\left(t\right)=\mathrm{AE}\left(t\right)$$

(3) $$\frac{\mathrm{du}\left(t\right)}{\mathrm{dt}}=A\frac{\mathrm{dE}\left(t\right)}{\mathrm{dt}}$$

and let

(4) $$E(t)=\bar{E}f(t)$$

with

(5) $$\left|f(t)\right|\;\leq 1\;\;\;$$

(6) $$\frac{dE(t)}{dt}=\bar{E}\frac{df(t)}{dt}$$

so that,

(7) $$i=C_{i}A\bar{E}\frac{df(t)}{dt}$$

where $\bar{E}$ is the strength amplitude of the near-electric-field generated from the DUT of magnitude *E*, *Ci* is the induced capacitance between the stripline of the DUT and the tip of the proposed probe, and *A* indicates the system constant. For the dominant electric field in the z-direction, we can express that the induced current can be approximately calculated as follows:

(8) $$E=E_{x}u_{x}+E_{y}u_{y}+E_{z}u_{z}$$

where *u_x_, u_y_*, and *u_z_* are unit vectors in the *x*, *y*, and *z*-directions, respectively.

Since

(9) $$E_{z}\;\gg \;E_{x}\;\;E_{z}\;\gg E_{y}\;$$

(10) $$i\;\approx C_{i}AE_{z\;}\frac{\mathrm{df}(t)}{\mathrm{dt}}$$

${\bar{E}}_{z}\;\;$ *f(t)* is the generated time-varying voltage in the z-direction between the microstripline of the DUT and the probe tip.

As shown in Figure 2b, the equivalent circuit for the E-field probe above the DUT gives the relation between the output voltage *V_O_* in probe port 2 and the input voltage *V_Z_* generated in port 1; it is the port assumed for the generation of the e-field, which can be expressed according to Kirchhoff’s Voltage Law (KVL) as follows:

(11) $$\frac{V_{0}}{V_{z}}\propto \frac{1}{1+\frac{C_{s}}{C_{i}}+\omega^{2}L_{s}C_{s}}$$

where *C_S_* is the induced shunt capacitance between the probe core trace and the ground plane of the proposed probe, *ω* is the working frequency, and *L_S_* is the generated self-inductance of the probe core trace.

To improve the probe performance, *Ci* must be as large as possible, so that the induced current *i* will be large and obvious; on the other hand, *C_S_* and *L_S_* must be as small as possible, so as to lead to a decrease in the leakage current *i_g_*, which will flow to the ground via the *C_S_* and *L_S_*.

At high frequencies, the problem will be obvious; so, increasing the effective area exposed to the incident *E_Z_* will increase the induced capacitance *Ci*, thus increasing the flowing current *i*. Additionally, decreasing the area of the ground plane relative to the probe tip will help to decrease the values of the shunt capacitance *C_S_* and the self-inductance *L_S_*. These parameters were carefully considered during the probe design process. The V-shaped structure of the probe tip plays a crucial role in enhancing and improving the performance of the E-probe.

### 2.3. Proposed V-Shaped Tip Structure

**Figure 3:**
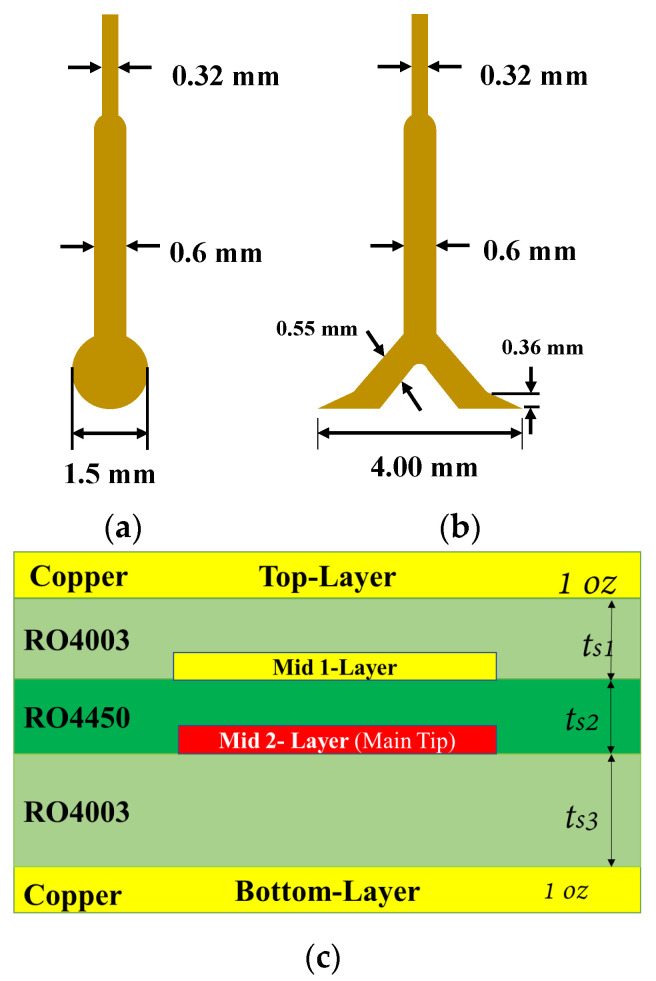
Tip structure. (**a**) Circle tip; (**b**) proposed V-shaped tip; (**c**) probe layers cross-section.

Related to Equations (10) and (11), the induced current will increase with the increase in the *Ci* so that the tip width is taken into account in the design. Figure 3 is a comparison between a pre-designed common circle tip structure and the new V-shaped structure; Figure 3b shows that the tip width in the proposed V-shaped structure is 4 mm, relative to the circle tip width in Figure 3a, which is 1.5 mm. This helps more induction charges to be induced in the probe tip. The two models in Figure 3 were built and simulated in HFSS under the same parameter conditions and the same four-layer layout that is illustrated in Figure 3c.

**Figure 4:**
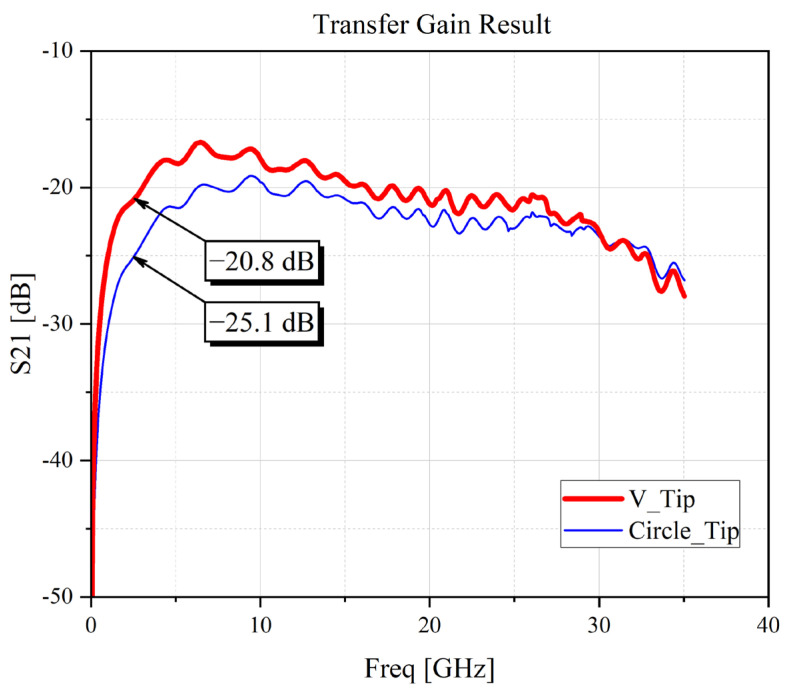
Simulation comparison result between V-shaped tip and circle-shaped tip.

The simulated result is shown in Figure 4 when the simulation is carried out at an angle 0° between the microstrip line and the probe tip. The simulation result illustrates the compatibility of the V-shaped design tip for the highest transfer gain, shown with a red line, which indicates a higher performance than the circle tip, shown with the blue line. The graph illustrates a higher 5 dB difference at a frequency of 5 GHz, and the difference is reduced smoothly and gradually to 2 dB up to 30 GHz.

### 2.4. Body Gradient and Edge Structure

The probe has been designed with a gradient edge structure, as mentioned in [16], which affects the probe transfer gain S21 at frequencies above 20 GHz. The gradient edge proves to be influential in minimizing the effects of abrupt structural changes, thereby establishing stable TEM transmission conditions. As a result, this design is anticipated to enhance the performance consistently throughout the entire frequency band.

**Figure 5:**
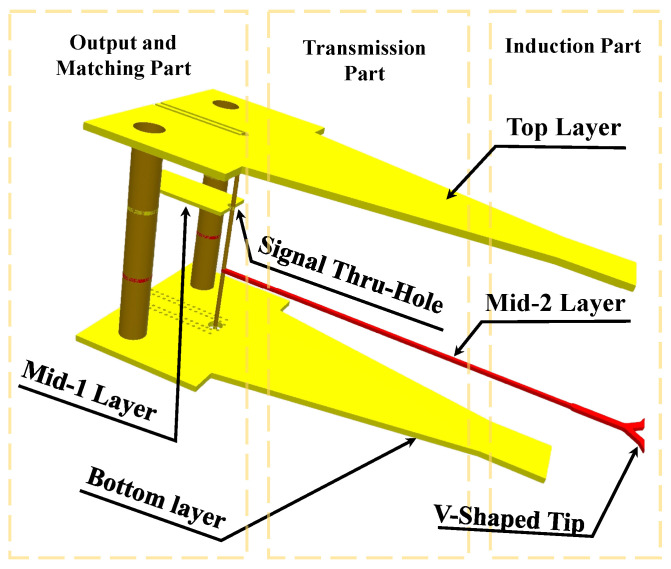
Simplified 3D structure of the proposed probe.

**Figure 6:**
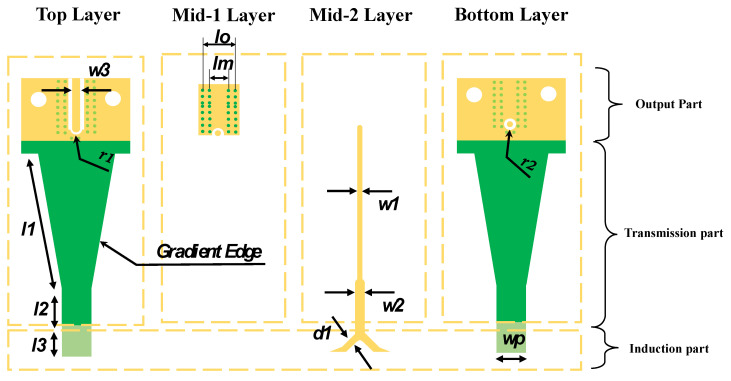
Simplified 2D structure of the proposed V-shaped E-probe.

The 3D and 2D views of the probe structure are shown in Figure 5 and Figure 6, respectively. The tuned size parameter values of the whole proposed structure are illustrated in Table 1; the upper and the bottom layers in addition to the probe sides are all covered with copper, which helps shield the noise and provides a good signal return path. In addition, to connect this probe to a receiver, we use a specially designed SMA connector, which can be used for very high frequencies up to 50 GHz.

## 3. Validation and Measurement

### 3.1. Frequency Bandwidth Measurement

The probe model simulation and design were built using HFSS, the calibrated device under test (DUT) is 50 mm × 50 mm, and the microstrip line width w = 1.16 mm. The DUT is a two-layer PCB with a substrate of Rogers RO4003 (tm), with εr = 3.55 and height (h) = 0.508 mm. As shown in Figure 2a, the probe is placed 0.3 mm above the microstrip line and perpendicular to the DUT surface, with an angle of 0° between the probe tip and the microstrip line. The probe is simulated from 10 kHz to 50 GHz with a sweep set to be interpolated with sweep points set at 1001 points.

**Figure 7:**
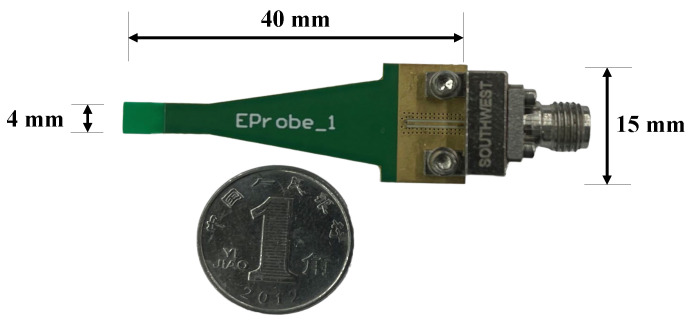
Photograph of the proposed passive E-probe.

**Figure 8:**
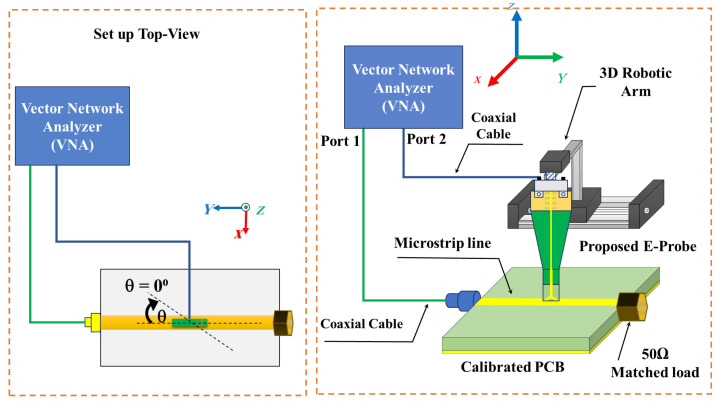
Proposed V-shaped probe measurement and orientation at an angle of 0°.

A photograph of the fabricated e-field probe is shown in Figure 7; the probe tip as shown in the photo is a compact size that allows for application in a variety of narrow and intricate spaces, making it compatible and adaptable for most types of PCB such as in avionics, UAVs, and electric trains. The working bandwidth measurement and validation setup is shown in Figure 8. The DUT is connected to port 2 of the vector network analyzer (VNA), and the PCB is excited with a frequency band of up to 60 GHz. A 50 Ω load matches the other port of the DUT. The probe is placed above the microstrip line, at 0.3 mm, and with an angle of 0°, and is then connected with the VNA port1.

**Figure 9:**
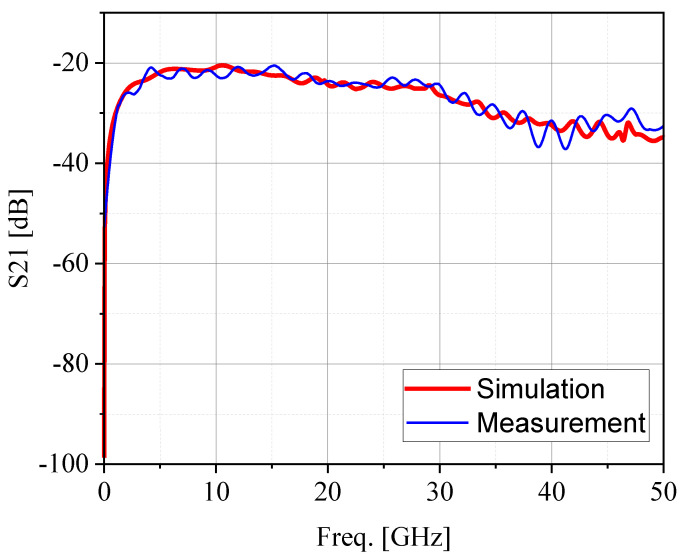
Transfer gain S21 between the simulation result and measurement result.

To validate the probe performance after fabrication, the comparison result is shown in Figure 9 between the simulation and the measurement result. From these results, it can be observed that the design performance and test performance of the 10 kHz to 50 GHz electric field probe are consistent up to 10 GHz. Above 10 GHz, both the simulated and measured performance exhibit a certain slope of attenuation, and the maximum error in the transmission coefficient is less than 5 dB up to 50 GHz. This difference is primarily due to the idealized nature of the simulation model, while the measured results consider the effects of probe fabrication processes and test environments.

### 3.2. Spatial Resolution

The spatial resolution can be defined as in [22]. The spatial resolution of the electric field probe is defined as the distance between the peak point and the −6 dB point in the near-field electric field distribution of the tested microstrip line, which is the spatial resolution at that test height. The spatial resolution is one of the main specifications and characteristics of the probes, which indicates the ability of the probe to distinguish between the components mounted inside a PCB and can pinpoint which component has a problem regarding the small distance between the components.

**Figure 10:**
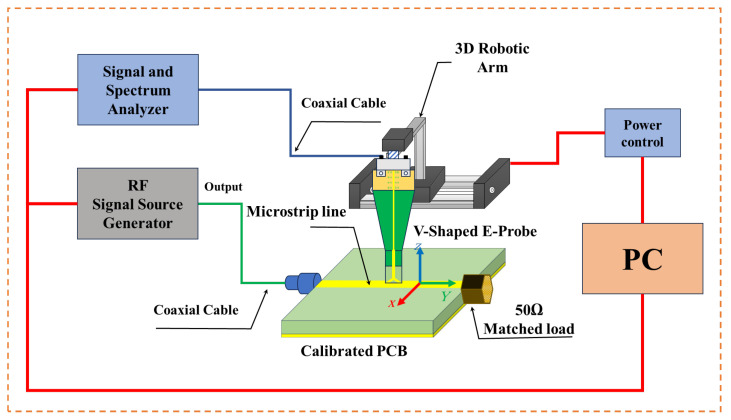
Spatial resolution test equipment connection diagram.

**Figure 11:**
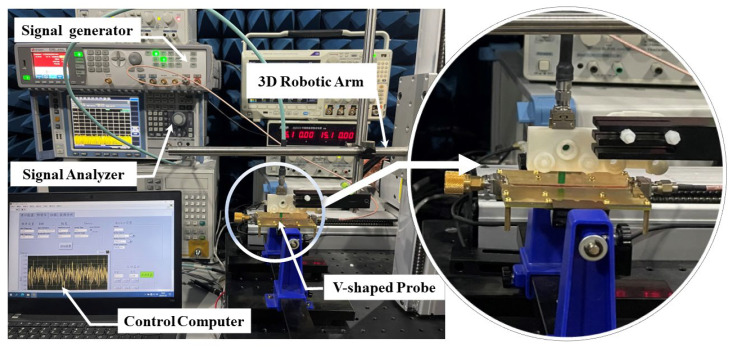
Spatial resolution test environment at the lab.

The measurement setup procedure and connection diagram for the spatial resolution is shown in Figure 10; the spatial resolution is measured, while the probe is placed 0.3 mm above the DUT, as shown in Figure 11, along the z-direction and with an angle of 0⁰. The probe is moved along the x-direction from −3 mm to 3 mm, with a step of 0.5 mm. The normalized transfer gain |S21| for the spatial resolution of the proposed probe is measured at 7, 9, 20, 25, 30, and 35 GHz, respectively.

**Figure 12:**
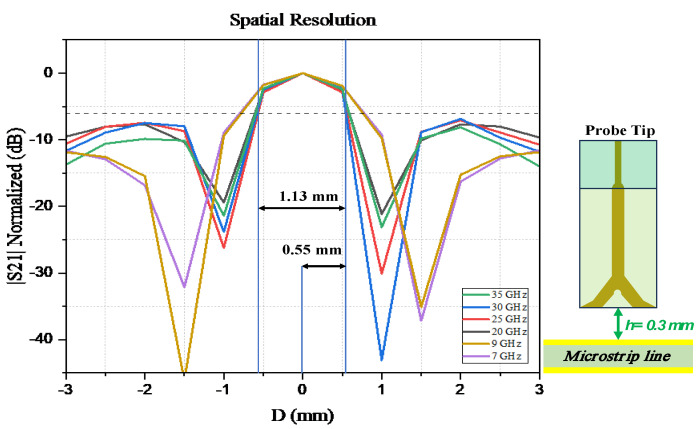
Spatial resolution results at 35, 30, 25, 20, 9, and 7 GHz.

The proposed probe’s spatial resolution is 0.55 mm at 30 GHz, and the result is very narrow compared with other referenced probes. The corrected normalized results of the spatial resolution of the electric field probe at different frequencies are shown in Figure 12. From the results, it can be seen that at a probe height of 0.3 mm from the DUT, the spatial resolution is approximately 0.55 mm.

### 3.3. Sensitivity

**Figure 13:**
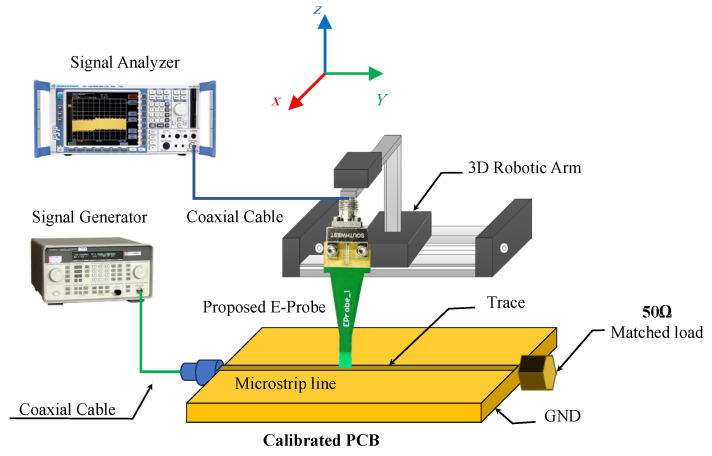
Simplified block diagram of sensitivity measurement setup.

The sensitivity is an important indicator of the electric field probe’s ability to measure weak signals. With the rapid development of science and technology, electronic devices often have low power consumption characteristics. This means that testing the near-field radiation characteristics of the tested devices becomes more challenging, and the sensitivity requirements for electric field probes are increasing. For the same tested target, an electric field probe with a higher sensitivity will produce higher output values. The electric field probe with a higher sensitivity can detect smaller amplitude electric field signals. The sensitivity setup process block diagram is shown in Figure 13; the sensitivity can be measured as follows: The device under test DUT is excited using the signal generator and matched by a 50 Ω load. The probe output port is connected to a signal analyzer. Then, when the probe tip moves near to center of the microstrip line trace, the output received signal by the probe is increased and can be plotted in the signal analyzer. When the received signal reaches a maximum, then start to decrease the output power of the signal generator up to a level that the received signal of the probe is submerged with the background noise of the test environment room. The output power then indicates the probe sensitivity.

The test measurement procedure of the sensitivity starts by exciting the DUT using a signal source (Agilent 8648C), using frequencies spanning from 10 kHz to 40 GHz. The probe is connected to a spectrum analyzer ROHDE&SCHWARZ, and the device background noise is set to be −130 dB. The minimum output power of the signal sources indicates the probe’s sensitivity for some different frequencies. Table 2 gives a comparison between the E-probe sensitivity and other referenced probes; the sensitivity performance of the proposed V-shaped tip probe is increased by 8 dB to 60 dB.

## 4. Application and Performance Comparison

**Figure 14:**
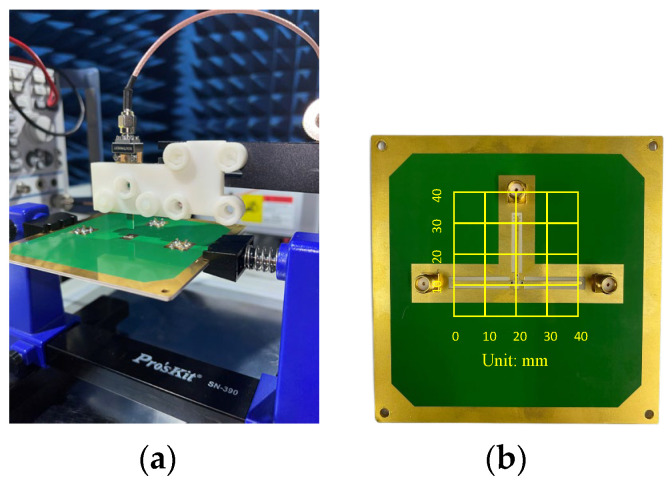
Near-field scanning: (**a**) amplifier PCB lab measurement scanning; (**b**) amplifier PCB.

The proposed E-Probe application and performance for near-field scanning have been performed on two different PCBs, which were used to indicate the performance of the proposed V-shaped tip probe in measuring, detecting, and pin-pointing the location of the EMI generated from different PCBs. The proposed probe is used for measuring and detecting the output e-field generated from two different PCBs, and the result is compared with another two probes—a circle-tip probe and a referenced horn-tip probe [6]. An amplifier PCB, as shown in Figure 14b, is one of the two PCBs used for evaluating the proposed probe performance. The amplifier PCB is excited by the power source, and it will generate a band of frequencies that the probe will detect. The scanning area is shown under the yellow grids of the amplifier PCB photo, and the probe is fixed as shown in Figure 14a, at about 2 mm above the PCB. The receiver is a signal analyzer that will detect the E_z_ field radiated from the PCB, and the background noise is defined at −110 dBm.

**Figure 15:**
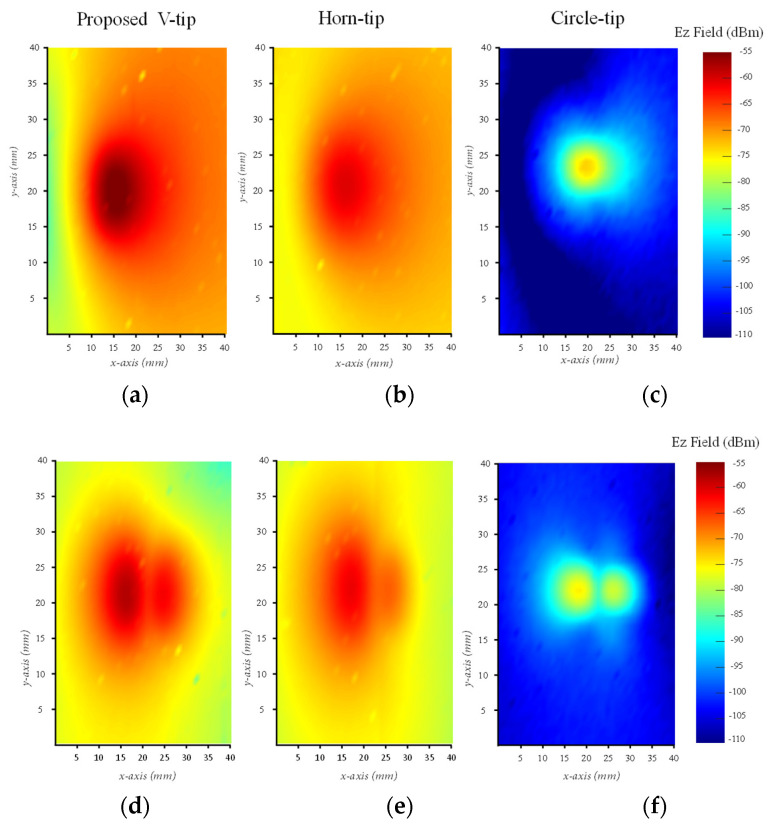
The near-field scanning result of two different PCBs: (**a**) received power of the proposed V-shaped probe for the amplifier PCB; (**b**) received power of the horn-tip probe for the amplifier PCB; (**c**) received power of the circle-tip probe for the amplifier PCB; (**d**) received power of the proposed V-shaped probe for the second PCB; (**e**) received power of the horn-tip probe for the second PCB; (**f**) received power of the circle-tip probe for the second PCB.

Figure 15a–c are the output received E_z_ power from the amplifier PCB and are detected by the three probes. Figure 15d–f are the received E_z_ power from the second PCB used in the evaluation test. The received detected power resulting from the three probes from the amplifier PCB and the second PCB are very clear.

In the amplifier PCB shown in Figure 15a–c, the maximum received and detected power using the proposed V-shaped tip probe is about −55 dBm, whereas the maximum detected power of the circle-tip probe is −73 dBm and that of the horn-tip probe in [6] is −68 dBm. In the second PCB shown in Figure 15d–f, the maximum power detected from the proposed V-shaped tip probe is about −55 dBm, while the circle-tip probe is −75 dBm and the horn-tip probe is −68 dBm. The mentioned received power is very clear and indicates the best performance of the proposed V-shaped tip probe design other than the circle-tip and horn-tip probes.

Table 3 presents the performance of the proposed V-shaped tip electric field probe in comparison to other referenced probes.

## 5. Conclusions

The fast development of electronic circuits and high-speed data transmission has led to the increased complexity and compactness of circuits. While these devices offer advantages such as a high efficiency, a compact size, and being lightweight, they also pose challenges related to electromagnetic compatibility (EMC). The radiation generated during their switching operation can introduce electromagnetic interference (EMI) and affect the performance of sensitive electronic systems. To address these issues, this article introduces a novel V-shaped tip probe design structure for an electric near-field measurement probe; the probe is small, reliable, ultrawideband to more than 50 GHz, and sensitive. The probe can be used to pinpoint, detect, and diagnose the electromagnetic interference (EMI) of any narrow and complex electronic device.

## Figures and Tables

**Table 1:** Tuning values of the proposed probe.

Parameter	Value (mm)	Parameter	Value (mm)
*l*1	20.3	*w*1	0.32
*l*2	5	*w*2	0.6
*l*3	5	*w*3	0.37
*lo*	2.4	*wp*	4
*lm*	1.4	*r*1	0.72
*d*1	0.55	*r*2	0.59
*ts*1	0.2032	*ts*3	0.3048
*ts*2	0.17		

**Table 2:** Proposed probe sensitivity comparison.

Freq.	Proposed	[15]	[23]	[24]
9 kHz	−15 dBm	13 dBm	10 dBm	16 dBm
1 MHz	−54 dBm	−20 dBm	−24 dBm	−15 dBm
100 MHz	−94 dBm	--	--	−50 dBm
500 MHz	−100 dBm	−40 dBm	−48 dBm	−64 dBm
1 GHz	−105 dBm	−82 dBm	−69 dBm	−69 dBm
5 GHz	−101 dBm	−82 dBm	−74 dBm	--
10 GHz	−100 dBm	−84 dBm	−75 dBm	--
20 GHz	−90 dBm	−82 dBm	--	--
30 GHz	−91 dBm	--	--	--
40 GHz	−87 dBm	--	--	--

**Table 3:** Performance comparison of the proposed V-shaped tip electric field probe.

Probe	Proposed	[15]	[25]	[26]	[27]	[22]
Tip	V-shaped	U-shaped	L-shaped	T-shaped	Rectangular	Monopole
B.W	10 kHz- 50 GHz	9 kHz- 40 GHz	10 MHz- 60 GHz	Narrow Band	6 GHz	9 kHz- 20 GHz
S21	−16 dB	−21 dB	−24 dB	−24 dB	−26.9 dB	−28 dB
Spatial Resolution	0.55 mm	1 mm	0.7 mm	2.5 mm	1.97 mm	2–3 mm
Detecting Field	Ez	Ez	Ez	Ez	Ez	Ez
Process	PCB	PCB	PCB	PCB	PCB	PCB

## Data Availability

Data are contained within the article.
